# A Secure and Robust Compressed Domain Video Steganography for Intra- and Inter-Frames Using Embedding-Based Byte Differencing (EBBD) Scheme

**DOI:** 10.1371/journal.pone.0150732

**Published:** 2016-03-10

**Authors:** Tarik Idbeaa, Salina Abdul Samad, Hafizah Husain

**Affiliations:** 1 Department of Electrical, Electronics and Systems Engineering, Faculty of Engineering and Built Environment, Universiti Kebangsaan Malaysia, Bangi, Selangor, Malaysia; 2 Department of Computer Science, Faculty of Science, Garyan University, Garyan, Libya; Kaohsiung Medical University, Kaohsiung 80708, Republic of China, TAIWAN

## Abstract

This paper presents a novel secure and robust steganographic technique in the compressed video domain namely embedding-based byte differencing (EBBD). Unlike most of the current video steganographic techniques which take into account only the intra frames for data embedding, the proposed EBBD technique aims to hide information in both intra and inter frames. The information is embedded into a compressed video by simultaneously manipulating the quantized AC coefficients (AC-QTCs) of luminance components of the frames during MPEG-2 encoding process. Later, during the decoding process, the embedded information can be detected and extracted completely. Furthermore, the EBBD basically deals with two security concepts: data encryption and data concealing. Hence, during the embedding process, secret data is encrypted using the simplified data encryption standard (S-DES) algorithm to provide better security to the implemented system. The security of the method lies in selecting candidate AC-QTCs within each non-overlapping 8 × 8 sub-block using a pseudo random key. Basic performance of this steganographic technique verified through experiments on various existing MPEG-2 encoded videos over a wide range of embedded payload rates. Overall, the experimental results verify the excellent performance of the proposed EBBD with a better trade-off in terms of imperceptibility and payload, as compared with previous techniques while at the same time ensuring minimal bitrate increase and negligible degradation of PSNR values.

## Introduction

During the past decade, the rapid advance of Internet, storage, digital communication technology, multimedia and network bandwidths technologies has enabled users to send digital data over network suitably. It is now possible to stream high quality video and to watch video online. The Internet and other digital networks are means for freely and widely distributing high fidelity of digital media [1]. However, the difficulties in ensuing individuals' privacy are also become increasingly challenging because transmission of data in an open network is not secure, and data can be easily tampered by unauthorized users. Consequently, shielding data during transmission is an important task.

Many researchers, companies, and organizations continue to find novel security measures that can provide better protection for secret information in terms of privacy, integrity, or authentication from being detected during transmission. Numerous research efforts in cryptography have been put forward, such as Data Encryption Standard (DES) [2], Rivest Cipher 4 (RC4) [3], Rivest-Shamir-Adleman encryption (RSA) [4], and Advanced Encryption Standard (AES) [5], where data are encoded into a form of cipher-text using a secret key.

However, such methods are not suitable for encrypting digital images without modification due to two strong reasons: characteristics of digital images and difficulties of distributing keys [6]. Moreover, cryptographic techniques are not secure enough because encryption can provide secure delivery of digital content during the transmission from sender to the receiver, but when the content is decrypted, encryption no longer provides any security [7]. Hence, to solve such problem, data hiding techniques are developed for such a particular multimedia application requirements, whereby the aim is to protect any kind of data, such as text, image, sound, and video, by hiding them into another cover media or carrier.

Data hiding techniques have been considered widely in various fields such as covert communication, copyright protection, broadcast monitoring and military communications. Steganography is defined as the art of imperceptibly hiding confidential information in such a way that no one realizes it except the sender and the authorized recipient. It is also known as ‘covert writing’ which includes methods of transmitting secret messages through inoffensive cover mediums in such a manner that the survival of the embedded messages is undetectable.

Visual data such as digital videos has become a popular multimedia content for both online and offline environments and therefore large video clips mean larger volumes of data can be embedded in video files but they require a large processing capacity or transmission bandwidth. Hence, they are often required to be converted in order to adapt to several channel capacities (e.g., network bandwidth) as well as end user's terminal capabilities [8].

Data hiding can be carried out in different domains. For instance, data embedded in spatial domain may be lost because of the lossy stage (quantization step) of the underlying video codec while in the frequency domain; data hiding is performed in the transformed coefficients where it survives the quantization loss but this may come at the cost of lower imperceptibility [9]. Some representative data hiding techniques in the video domain could be found in [10–18]. For example, Nakajima et al. proposed a rewritable data embedding method on MPEG coded data domain.

Data embedding is performed in a block by block basis utilizing the length of zero-run coding value of AC-QTCs as a host signal. In this method, data bits are embedded into I-, P- and B-frames and the data payload is controlled by adjusting a certain parameters thereby achieving low embedding capacity. The results demonstrated that the payload size in I- and P-frames is much larger than of B-frames [10]. Xu et al. proposed steganography method that operates directly in compressed bit stream. The method embeds data bits into both inter- and intra-frames by modifying coefficients at selected locations in each 16 × 16 block within 330 macroblocks (MBs) of each frame. Furthermore, the data bits are repeatedly hidden in motion vectors (MVs) of MBs that have larger motion speed in order to resist video processing. The maximum embedded data payload was only about 179 bpf for I- and P-frames and it was about 564 bpf for B-frames. Even though the embedded payload capacity is low, the quality of the stego frames are significantly distorted where the obtained PSNR values of 35.22 dB,34.61 dB,33.31 dB are reported for I-, P- and B-frames respectively [11].

For low bitrate increase due to data embedding, Wong et al. [12] proposed a data hiding scheme that completely preserves the size of the video file using the scaling factor in rate controller as the data carrier. As result, no bitrate increase has been reported and the stego video has been generated with exactly the same bitrate as the original host signal (i.e compressed signal without data embedding). Sarkar et al. proposed high-volume transform domain data hiding scheme in MPEG-2 video sequences. The scheme is based on applying the quantization index modulation (QIM) mechanism to the selected set of DCT coefficients with low-frequency. The QIM is an adapted quantization parameter according to MPEG-2 parameters. Furthermore, the embedding payload rate is varied depending on the type of frame; hence, each frame is employed separately. Since insertions and deletions processes occur at the decoder, this may cause some problems such as de-synchronization and decoding failure. Such problems are solved by utilizing powerful turbo-like codes and erasures at the encoder side [13].

Wang et al. proposed a data hiding scheme to embed information into MVs of P-frame of the compressed video. In this scheme, the embedded information is extracted from every region of utilized MVs during the process of the decoder. The results of the scheme demonstrated that there was little impact on both video quality and the bit rate increase where an average increase of 4% in the video bit rate has been reported [14]. To completely preserves the quality of the host video sequence, Wong et al proposed a reversible data hiding scheme that embeds information into I-, P- and B-frames of the compressed video domain by manipulating the quantized coefficients (QTCs). Furthermore, to improve the embedding efficiency, the scheme utilized a new proposed representation mechanism namely reverse zero-run length (RZL) and it is evaluated over various MPEG-1 compressed videos. It is theoretically and experimentally verified that the RZL provides good performance in terms of embedding capacity and embedding efficiency for this particular embedding scheme. However, an average increase of 4-bits in the video file size is observed for every data bit embedded [15].

To enlarge the payload rate of the embedded data, Sherly and Amritha proposed a compressed video steganographic scheme namely tri-way pixel-value differencing (TPVD).The TPVD scheme operations are executed entirely in the compressed domain of MPEG standard. The scheme is tested on I-frame with maximum scene change and on P- and B-frames with maximum magnitude of MVs. Experimental results demonstrate that intra frames yields the highest payload and imperceptibility compared to inter frames (i.e. P- and B-frames) [16]. However, a high percentage modification in QTCs will certainly lead to significant distortion in the generated stego video.

Ali et al proposed compressed video steganographic scheme based on the concept of differential expansion (DE). The scheme utilized both luminance and the chrominance components within the intra pulse code modulation macroblocks of the P-frames of the H.264/AVC. It was tested for a variety of video sequences and compared to other techniques having similar concept. The results demonstrated that the proposed scheme provides better performance in terms of embedding capacity, lower bitrate increase compared to other techniques and negligible degradation of video quality [17].

Comparative Analysis of various data hiding techniques in Compressed Video Domain was presented by Tarik et al. [18]. Data hiding techniques were analyzed in terms of effectiveness in concealing data within the MPEG-2 compressed video. Three embedding techniques, namely, least significant bit insertion (LSB) proposed by [19], bit-plane complexity segmentation (BPCS) proposed by [20], and enhanced version of pixel value difference (EPVD) proposed by [21], were implemented and analyzed using a different performance metrics. Furthermore, Data embedding was performed on the AC-QTCs of luminance components of the I-frames during the MPEG-2 compression process. The results show that, among the analyzed techniques, EPVD provided better trade-off performance between imperceptibility, capacity, and level of security compared to its original version proposed by [22]. This is because the EPVD avoids the visible artifacts that could be produced from embedding a large amount of data while simultaneously providing slight security.

Even though the above-mentioned data hiding schemes generally produce an acceptable degree of video quality regardless of their applications and data carrier in use, the overall imperceptibility, of the modified video (i.e. stego video) is always limited to embed data payload. This is a critical drawback because these data hiding technologies cannot be utilized in applications where degradation in the quality of video sequences is not allowable. To solve this problem, we propose a novel data hiding technique (i.e. Embedding-Based Byte Differencing (EBBD)) in the compressed video domain that completely preserves the video quality based on the main steganography issues, such as imperceptibility, embedding capacity, and level of security and archives better trade-off between these issues.

## Video MPEG-2 Compression

Due to the huge amount of the data present in motion video, the MPEG-2 is designed as a compressed digital video standard which removes both the temporal and spatial redundancy. Further reduction of the redundancy data is achieved by run length and variable length coding. The MPEG-2 is split into hierarchy layers startly with video sequence layer and endly with block layer. The video sequence layer consists of a number of group of pictures (GOPs). The frames of each GOP in this study are independently encoded and decoded. Usually, the GOP structure consists of three types of pictures: intra-frame (I-frame), bidirectional-frame (B-frame), and the predictive-frame (P-frame) and the GOP may take different length (i.e. composed of one I-frame, some B-frames and, possibly some P-frames).

Generally, the maximum GOP length depends on the specifications of the playback device whereas the minimum GOP length depends on the GOP pattern. Although small GOP patterns with a short GOP length do not compress the data rate as effectively as large GOP patterns with a long GOP length, it works better with videos that have quick movements. In other words, longer GOP lengths encode video more efficiently by reducing the number of intra frames.

However, a long GOP is less desirable for short-duration effects, such as fast transitions or quick camera pans. Furthermore, a long GOP means that several inter frames (i.e. B-frames) are used between intra frame intervals, providing high image quality for a given data rate but requiring a long time to decompress. This result happens because B-frames within the GOP are reliant on frames that may be reliant on other frames in addition to the P-frames that are dependent on the I-frames for referencing.

For most encoders, the default GOP structure is a 12-frame GOP, which is IBBPBBPBBPBB before the repeat. However, if the video is detailed or fast moving, then it can be shortened to a six-frame GOP or less, especially in the case of real-time transmission of MPEG video in which the GOP structure is typically composed of six frames patterned as IBBPBB. Consequently, the process of adjustment in the GOP structure may result into either increases or decreases the resolution, encoding and decoding time and compression performance.

Therefore, a short GOP structure with the length of six and display order of IBBPBB is chosen to be used for implementing the proposed video steganography system. Each of these pictures is broken up into *M* number of slices where each slice is further broken up into *N* numbers of macro-blocks (MBs) and each MB has four 8 × 8 luminance blocks (Y) and two chrominance blocks (U and V). The content of each block is a 2-D DCT coefficient that is quantized using uniform scalar quantizer (USQ) specified by a fixed quantization table which is then variable length coded (VLC). Given that Y-component (brightness) blocks are located in an unaffected channel; these are used as host blocks to minimize the color distortion in the embedded video.

## Secure EBBD Steganographic Algorithm

### System Overview

This study devised a complete MPEG-2 video data hiding scheme. Data are embedded in the luminance space during compression of a CIF file. Considering the standard color space of video and low correlation among three color components, YCbCr color space is used in the proposed video data hiding scheme. Conventional videos consist of a number of GOPs; in this paper, the GOP structure consists of six pictures in display order "IBBPBB,". The frames of each GOP are independently encoded and decoded.

Each frame is divided into N numbers of slices consisting of M macro-blocks, and each macro-block has four 8x8 luminance blocks (Y) and two chrominance blocks (U and V). The 2-D DCT is applied to each non-overlapped 8×8 block in 64 DCT coefficients. These coefficients are then quantized using uniform scalar quantizer, specified by a fixed quantization table, zigzag scanned, and VLC coded. Since the Y-components (brightness) blocks are on an unaffected channel, they are used as host blocks to minimize the color distortion in the embedded video.

The alteration of a single coefficient affects the entire 64 pixels block, but because the change works in the compressed domain rather than the spatial domain, there will be less visual artifacts in the reconstructed frame if those coefficients are handled carefully. For the purpose of avoiding creating step effects in block coefficients that increase the distortion to the stego-signal, the QTCs should be modified as little as possible. Furthermore, the AC-QTCs become zeroes mostly after quantization. These zeroes are usually sensitive of embedding and the embedding capacity of the compressed host signal. Indeed, a high percentage modification in AC coefficients will certainly lead to significant distortion in the generated stego-signal. Therefore, an optimal balance between degradation and embedding capability should be determined.

### Host Coefficients Selection Mechanism

To embed data bits into MPEG-2 compressed video, an ordinary presentation mechanism namely, HCSM is proposed in order to select the proper AC-QTCs as carrier. For every desired frame, *F*_*x*_, let *B*_*q*_ refer to each 2D-QTCs 8×8 block and *B*_*z*_ is the output of the zigzag scan operation, *B*_*z*_ = {*C*1,…,*C*64}, where *C*1 is the DC coefficient and *C*2,…,*C*64 are the AC coefficients. Therefore, a subset of coefficients is extracted from *B*_*z*_ containing 63 AC coefficients is denote as *B*_*s*_, $B_{s}=\left\{C_{AC_{1}},\ldots \;,C_{AC_{m}}\right\}\subset B_{z}$, where *m* < 64 and are considered suitable coefficients for data hiding. Since the frame size is 352 x 288 pixels, this resulting in a total of 1584 of 8 x 8 DCT blocks, *T*_*Bs*_ = 1584 where *T*_*Bs*_ is computed as

$$T_{BS}=\left(\frac{Width\;of\;F_{x}}{8}\right)\times \left(\frac{Hight\;of\;F_{x}}{8}\right)$$ (1)

These *B*_*s*_ subsets are then arranged in 2-D arrays denoted as $M_{SB}$, in which each row in this matrix represents the AC-QTCs of each *B*_*s*_. Hence, the $M_{SB}$ is used as the host signal for carrying the secret information bits. Once all data bits are successfully embedded, each *B*_*s*_ in the modified array, $M_{SB}^{\prime }$ will be organized with the corresponding DC coefficient to get the entire 64 coefficients and are then subjected to VLC coder.

### Embedding Strategy

**Fig 1:**
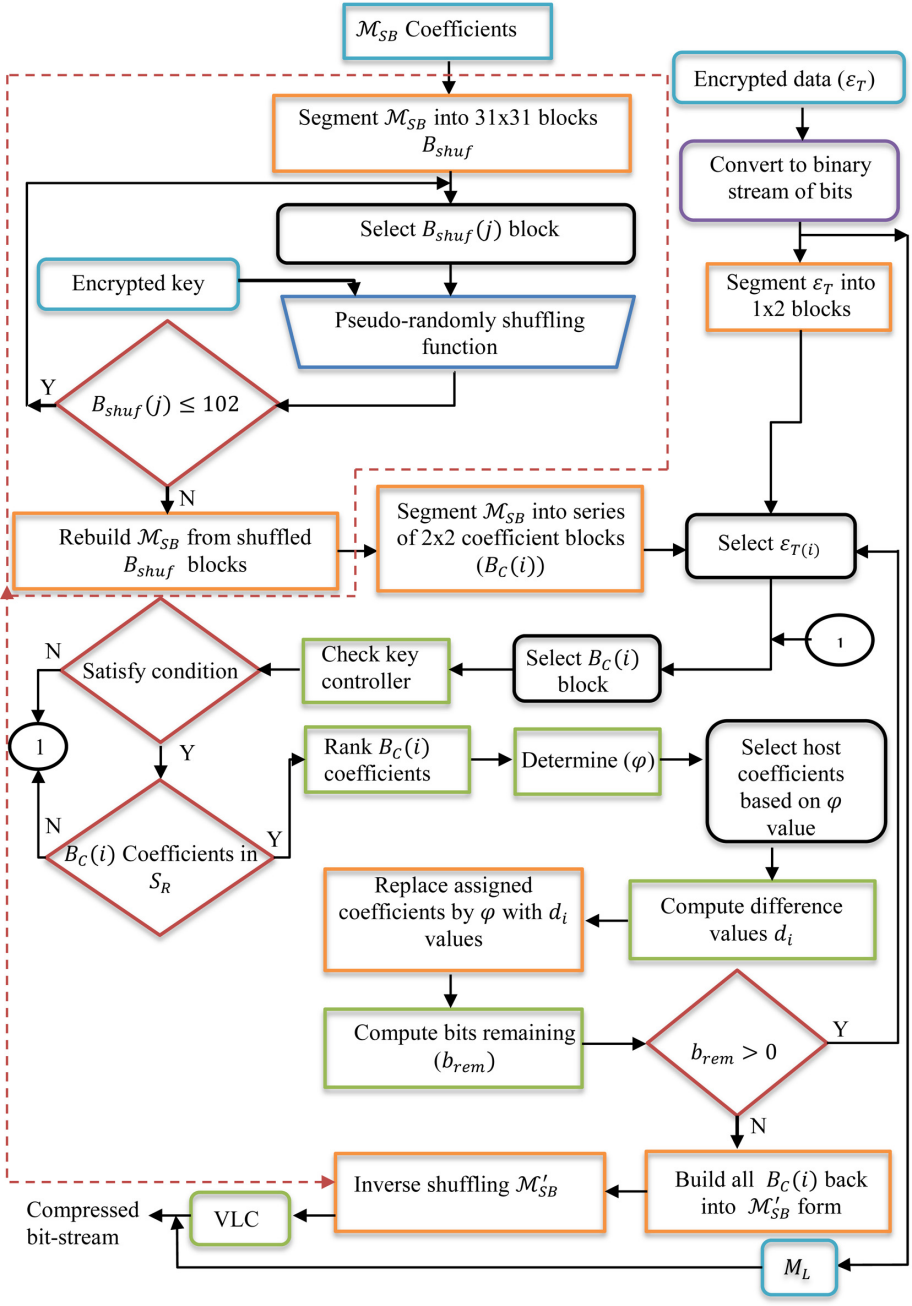
Flowchart for data embedding in the compressed domain using EBBD steganographic scheme.

The data path and flowchart of the data embedding process using the EBBD steganographic algorithm in the compressed MPEG-2 domain is shown in Fig 1. Similarly, encrypted text data are embedded into the Y color space only. To further clarify the illustrated flowchart figure, the steps involved in data embedding using the proposed EBBD method are described in the following subsections.

#### Security analysis

This work basically deals with two security concepts for the embedded data from being detected: Data are encrypted and the randomness of the selected host AC- QTCs. The concept of encrypting data has already been achieved by using S-DES technique [23]. The randomness concept is based on shuffling the host coefficients ($M_{SB}$) before initiating the embedding phase. Knowing the size of $M_{SB},\;Z_{M}=1584\times 63$ which is determined by Eq 1, means that there are 1584 blocks of coefficients each with size of 63. Before shuffling the coefficients, the $M_{SB}$ segmented into 31×31 blocks denoted as *B*_*shuf*_ (*j*), *j* = (1,…,*h*), where *h* is the total number of selected blocks is computed using Eq 2 and is equal to 102 blocks. The total number of the blocks and size of each block have been chosen based on the largest possible fraction for a block size from a given $M_{SB}$ size.

$$h=\left(\left(T_{Bs}-3\right)\times \left({\text{B}}_{\text{s}}-1\right)\right)/\left(31\times 31\right)$$ (2)

**Fig 2:**
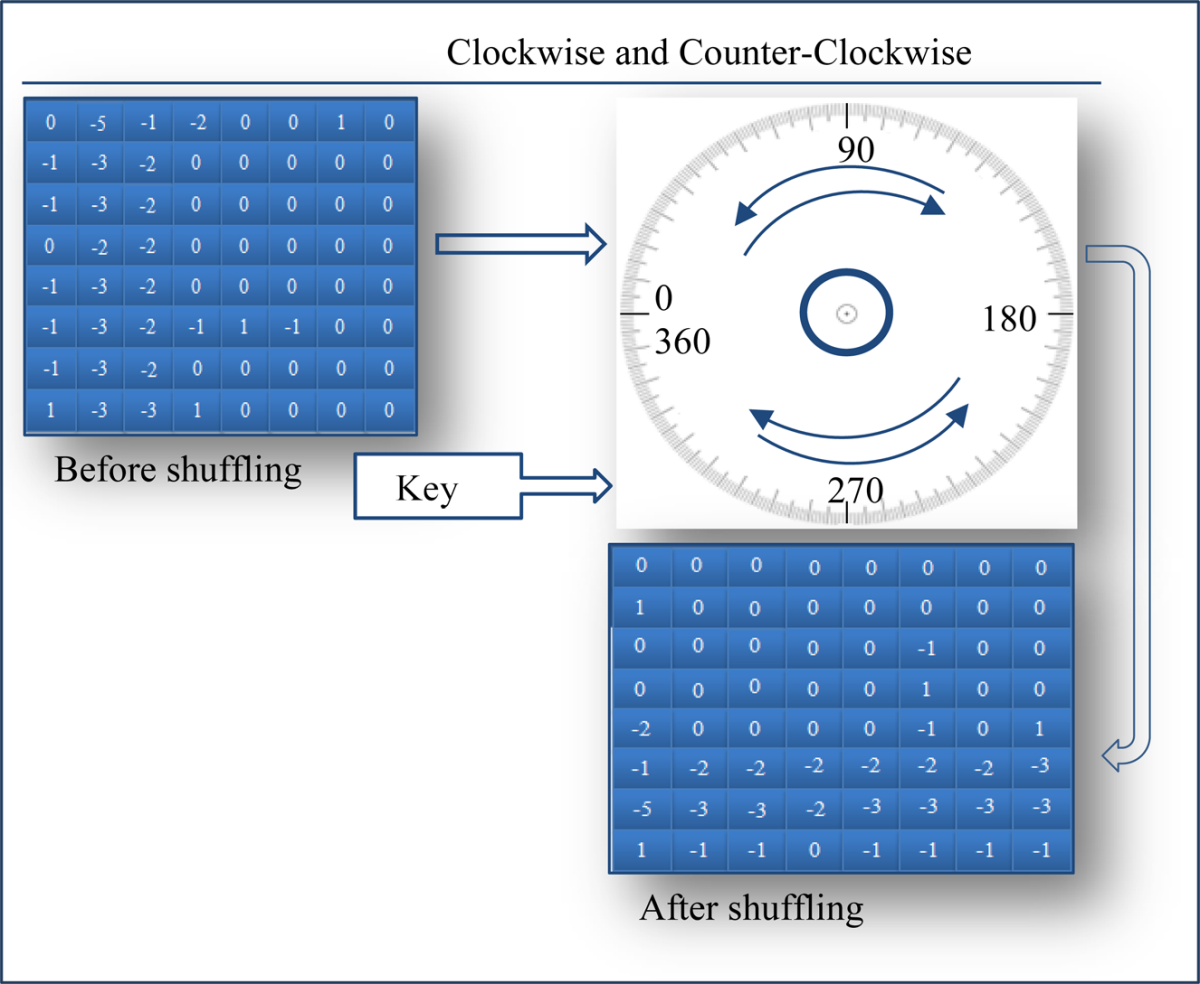
Example of an 8x8 block of coefficients before and after applying pseudo-randomly shuffling technique.

These blocks are then randomly flipped by 0°, 90°, 180°, 270°, and 360° as shown in Fig 2 using a key. For additional layer of security, the key is encrypted using the same encryption algorithm that is used for encrypting data. Furthermore, this key can be encrypted using either symmetric algorithms, such as in [2, 3], or asymmetric algorithms such as in [4, 5]. Asymmetric algorithms have certain advantages over symmetric algorithms, where a pair of different keys is involved, namely, public and private keys. Therefore, asymmetric algorithms rely on various mathematical computations, which make these algorithms slower than symmetric ones.

By contrast, asymmetric algorithms are computationally more expensive and time consuming, apart from having significant practical difficulties in securely distributing a symmetric key. Since both symmetric and asymmetric algorithms are used for secret communication but not for concealing communication and to avoid an expensive processes and time-consuming, the S-DES, regardless of the use of such algorithm to encrypt confidential data, has been used as further method to provide an additional layer of security to the embedded data by encrypting the secret key itself that is used to pseudorandomly shuffle the coefficients in each segmented block of coefficients. Thus, it is virtually impossible for unauthorized people who know the algorithm to pirate the hidden information, without knowledge of the encryption key.

Hence, shuffling implies that the AC- QTCs of each row (*B*_*s*_) in $M_{\text{SB}}$ now come from different random parts of a frame. These coefficients are then subjected to embedding process and once all the data bits have been embedded, the inverse shuffling phase for the coefficients in $M_{\text{SB}}^{\prime }$ is processed using the same encrypted key.

#### Pre-processing phase

**Fig 3:**
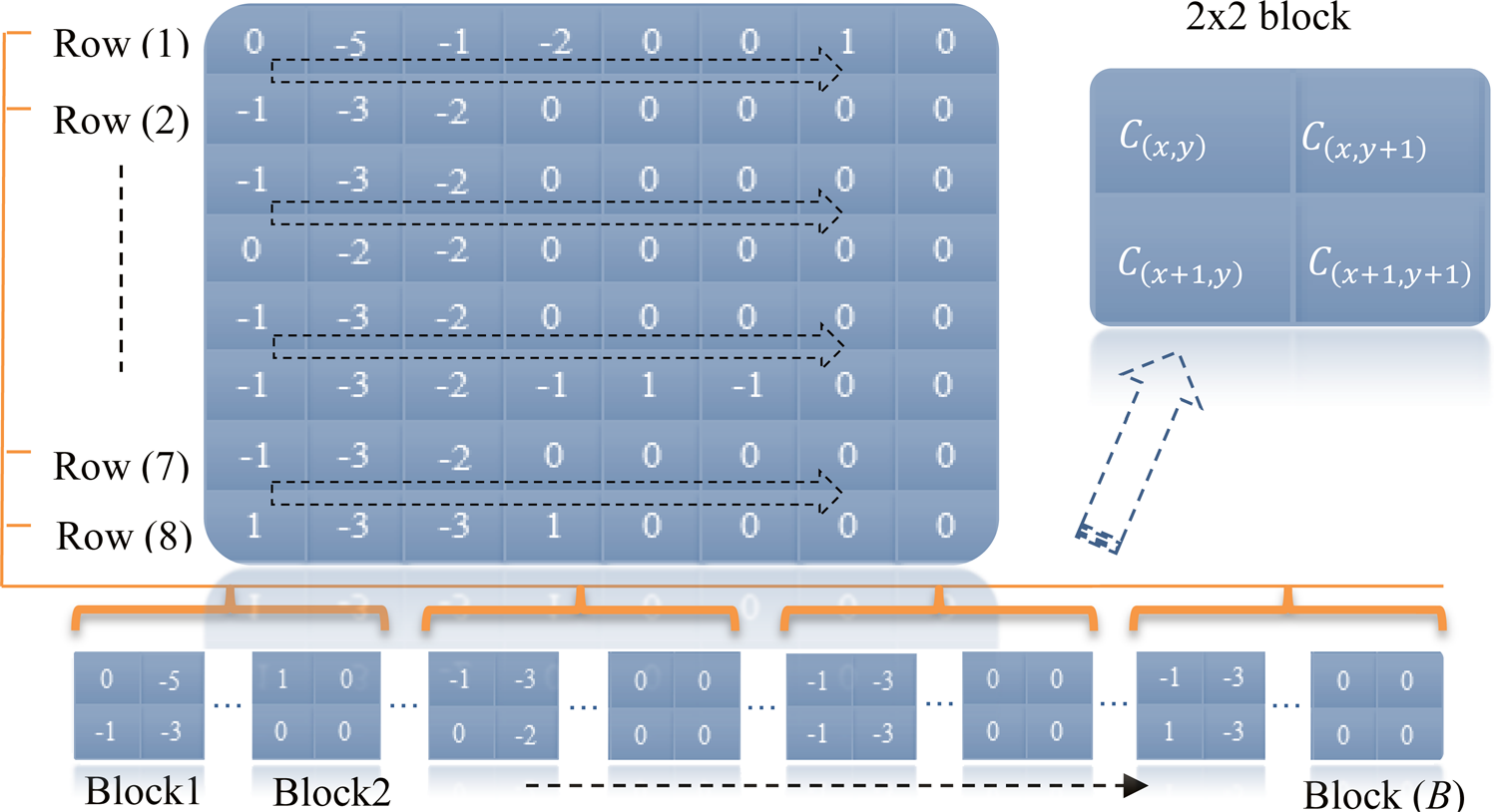
Example of segmenting 8×8 coefficients data into a series of 2×2 blocks.

In the proposed steganographic scheme, the host $M_{SB}$ coefficients is portioned into series of non-overlapping 2 × 2 blocks denoted as *B*_*C*_(*i*), where *i* is the block index, by running every two rows by two rows respectively from $M_{\text{SB}}$ in a survey sequential manner as shown in Fig 3. Each *B*_*C*_(*i*) is the basic structure in the embedding scheme in which the four coefficients in each block are denoted as *C*_(*x*,*y*)_, *C*_(*x*,*y*+1)_, *C*_(*x*+1,*y*)_ and *C*_(*x*+1,*y*+1)_ where x and y represent the location of the coefficient in the block. However, the size of $M_{\text{SB}}$ has to be any even number and an odd number is not allowed because the pattern cannot contain pairs of coefficients. Since each vector of the AC coefficients, $R_{k}\left(\text{v}\right)$, in the $M_{\text{SB}}$ is of 63coefficeints, only 62 coefficients out of 63 are used for embedding data and the last coefficient is discarded. Therefore, the host signal of the AC coefficients is defined as:

$$M_{SB}=\left[\begin{array}{c} R_{1}\left(v-1\right)\\ \vdots \\ \vdots \\ R_{k}\left(v-1\right) \end{array}\right]\subset F_{x}$$ (3)

The technique determines the QTCs that can be used for embedding using two different branch conditions in order to avoid undesired visual distortion in a stego-signal. The first condition determines the embeddable and non-embeddable blocks of quantised DCT coefficients while the second condition identifies the coefficients within the determined blocks to be used for embedding. The *C*_(*x*,*y*)_ Coefficient is chosen to be used as the key controller parameter for making a decision of whether the current selected block, *B*_*C*_(*i*), is suitable or not for data embedding. The recommended choice for the key controller parameter value is, when the *C*_(*x*,*y*)_ value is between "0" and "2" and blocks with *C*_(*x*,*y*)_ value not among all possible values that satisfy the requirements of this technique are discarded. The non-chosen blocks and their AC coefficients remain the same. Based on the first condition; the suitable host *B*_*C*_(*i*) can be expressed as

$$B_{C}\left(i\right)=\left[\begin{array}{cc} C_{\left(x,y\right)} & C_{\left(x,y+1\right)}\\ \; & \;\\ C_{\left(x+1,y\right)} & C_{\left(x+1,y+1\right)} \end{array}\right]\;,\;0\;\leq C_{\left(x,y\right)}<3$$ (4)

Thus, the maximum probabilities of embedding data bits are only in three coefficients; that is, only three of four AC- QTCs values can be selected to embed data. Each value of these coefficients is located in selected coefficients range (*S*_*R*_), *R* = (2, 1, 0, -1, -2) 3 > *S*_*R*_ > −3, because a large coefficient value will degrade the quality of the stego-frame. Thus, only possible coefficients selected within the allowable range in this technique guarantee the distortion would be unnoticeable to the human eye.

**Fig 4:**
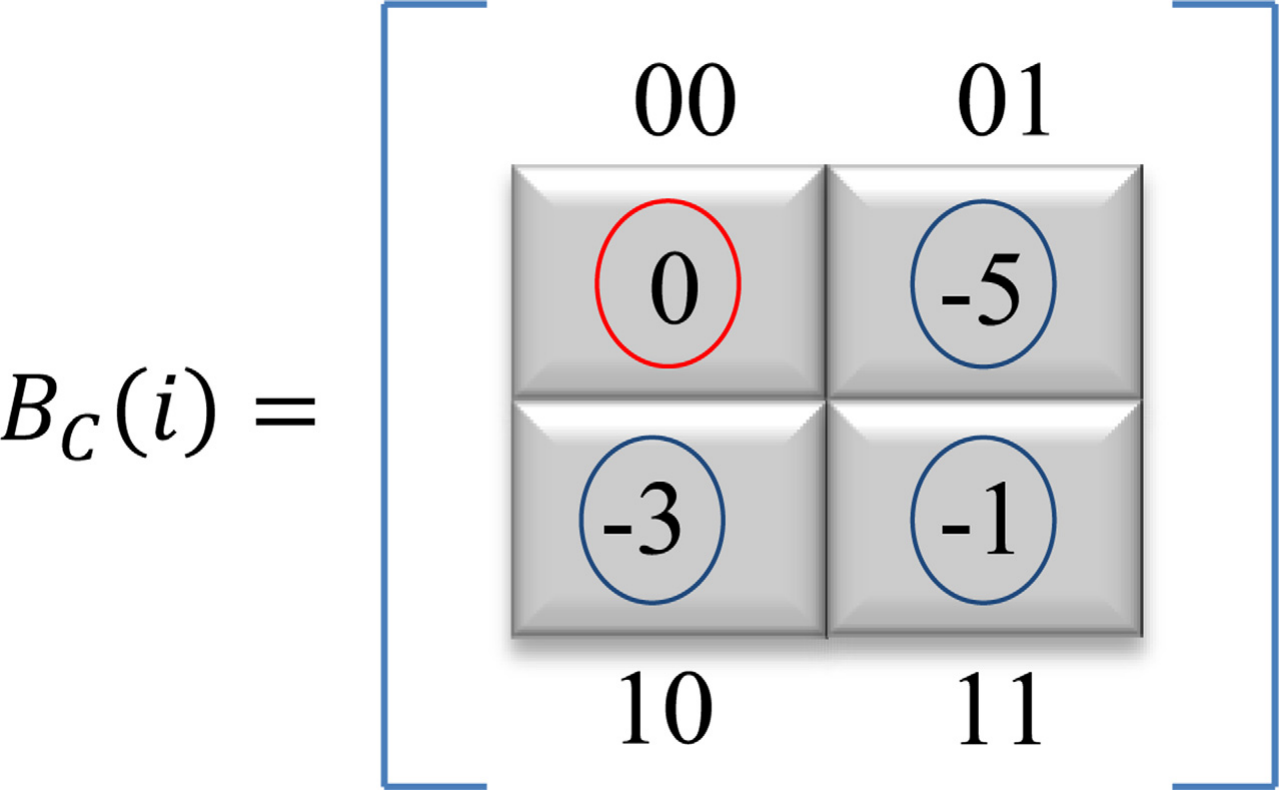
Ranking the coefficients locations for each *B*_*C*_(*i*) block.

To identify the coefficients within the determined blocks to be used for embedding, each of the embeddable block is sequential ranked denoted by, *β* = {*β*_0_,*β*_1_,*β*_2_,*β*_3_}, Where *β*_0_ = (00), *β*_1_ = (01), *β*_2_ = (10), and *β*_3_ = (11). Fig 4 explained the ranking method of coefficients location for each these blocks.

$$B_{C}\left(i\right)=\left[\begin{array}{cc} C_{\left(x,y\right)}^{\beta_{0}} & C_{\left(x,y+1\right)}^{\beta_{1}}\\ \; & \;\\ C_{\left(x+1,y\right)}^{\beta_{2}} & C_{\left(x+1,y+1\right)}^{\beta_{3}} \end{array}\right]$$ (5)

An indicator (*φ*) is produced to decide which coefficients are selected for future use. The XOR operator, which is performed between the key controller value *C*_(*x*,*y*)_ and the decimal value of the highest AC coefficient ranking, determines the value of this indicator. Thereafter, the modulo operation is applied to the previously obtained results and the total number of the allowable AC coefficients, *Γ*, to determine the value of *φ*.

$$\varphi =\;\left(C_{\left(x,y\right)}⊕{\left(\beta_{3}\right)}_{10}\right)\;\text{mod}\;\Gamma$$ (6)

In this category (*Γ* = 3), the following three cases are considered according to the value of *φ* to identify host coefficients.

$$\varphi \;=\;\{\begin{array}{l} 0\;,\;\left(\beta_{1},\beta_{3}\right)\\ 1\;,\;\left(\beta_{1},\beta_{2}\right)\\ 2\;,\;\left(\beta_{2},\beta_{1}\right) \end{array}$$ (7)

Therefore, the host coefficients pair will be selected according to the value of *φ* as following:

Case 1: The coefficients mapped as (*β*_1_, *β*_3_) are selected if *φ* = 0.
Case 2: The coefficients mapped as (*β*_1_, *β*_2_) are selected if *φ* = 1.
Case 3: The coefficients mapped as (*β*_2_, *β*_1_) are selected if *φ* = 2.

The bytes of the encrypted secret data (ε_T_) are broken up into a series of blocks, (*ε*_*T*(*i*)_), *i* =1,…,*R* where *R* is total number of secret data blocks and ε_T(i)_ ∈ {0,1}. Each block consists of two bits. These bits are transformed individually into decimal value denoted by *ε*_(D)_, *ε*_(D+1)_ where *D* represents the location of the bit in the secret data block. The proposed scheme permits embedding two bits into two coefficients within *B*_*C*_(*i*) by determining the difference between the value of key controller parameter and each transformed secret data block. This difference is considered as the modified coefficient value and replaces the original coefficient value that was selected according to *φ*. The equation below, which is built up in accordance with the HVS features to guarantee the stego-signal still preserves good quality, confirms the probability of the information embedded in the corresponding coefficient.

$$d_{i}=[C_{\left(x,y\right)}-{\varepsilon_{\;\text{T}\left(\text{i}\right)}]|}_{\varphi =0,1,2}$$ (8)

The two corresponding coefficients are expected to carry *ε*_*T*(*i*)_ bits; that is, the coefficient values are changed to the absolute value of their new difference. The embedding scheme changes some of the coefficient values closer to zero and some values further. Accordingly, the average bit rate remains more or less the same. For simplicity, according to *ε*_*T*(*i*)_, two possible differences are determined through the following equations:

$$d_{1}=\left(C_{\left(x,y\right)}-E_{\left(\text{D}\right)}\right)$$ (9)

$$d_{2}=\left(C_{\left(x,y\right)}-E_{\left(\text{D}+1\right)}\right)$$ (10)

Therefore, the modified block $B_{c}^{\prime }\left(i\right)$ of the host signal $M_{\text{SB}}$ can be expressed as:

$$B_{c}^{\prime }\left(i\right)\;=\;\{\begin{array}{l} \;\begin{array}{ll} C_{\left(x,y\right)} & d_{1}\\ C_{\left(x+1,y\right)} & d_{2} \end{array}\;,\;if\;\varphi =0\\ \;\begin{array}{ll} C_{\left(x,y\right)} & \;d_{1}\\ \;d_{2} & C_{\left(x+1,y+1\right)} \end{array}\;,\;if\;\varphi =1\\ \;\begin{array}{ll} C_{\left(x,y\right)} & \;d_{2}\\ \;d_{1} & C_{\left(x+1,y+1\right)} \end{array}\;,\;if\;\varphi =2 \end{array}$$ (11)

**Fig 5:**
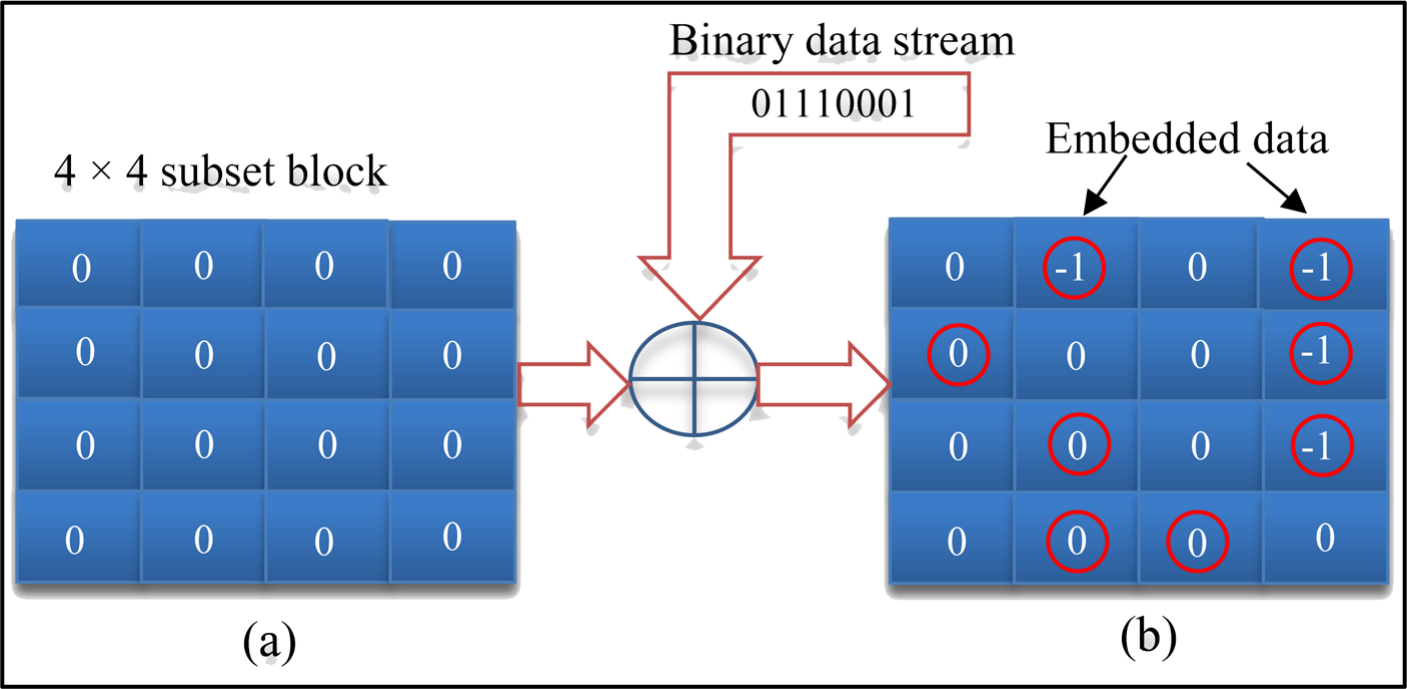
An example of selected 4x4 sub-block of AC- QTCs with embedded data. (a) Before embedding and (b) after embedding.

The advantage of this strategy is that all of the AC- QTCs with zero values can be used for embedding data in the compressed video domain while maintaining high perceptual quality. Fig 5 shows an example on how to convey eight bits of secret data into a 4 × 4 subset block of AC- QTCs of a selected block of *B*_*C*_(*i*). According to experiments, a choice of two AC- QTCs can provide excellent visual quality. In addition, three bits of data can also be hidden in three AC coefficients by modifying the technique. As can be seen from Fig 5 below, only four coefficients have been modified by a value of “-1” due to the secret bit value while the rest of the coefficients remains the same.

#### Extraction strategy

**Fig 6:**
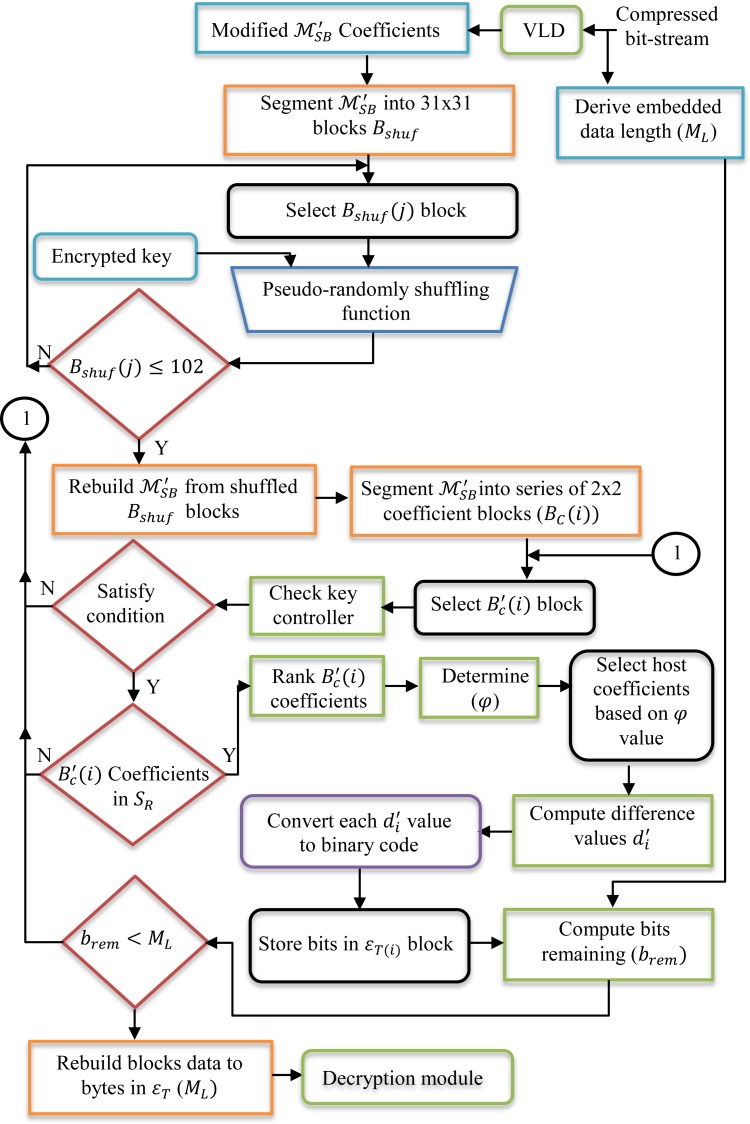
Flowchart for data extraction in the compressed domain using EBBD steganographic scheme.

To retrieve the embedded data, the modified video is parsed in the exact same order as a data embedder does. The flowchart for data extraction is shown in Fig 6. The initial steps of the extraction procedure are similar to those of the embedding process and host coefficient selection. Embedded data are extracted before the frame is fully decoded while ignoring the quality of the decoded frame. In this step the embedded secret bits are retrieved by computing the difference between the value of key controller parameters and modified coefficients (which are the values of differences *d*_*i*_). Since the maximum probabilities of embedding data bits is only into three coefficients, only three out of four different coefficients are identified within a selected block *B*_*C*_(*i*) using Eqs (6) and (7). Therefore, according to *φ* value, the modified coefficients are selected and the difference value $d_{i}^{\prime }$ between the key controller parameter value and each of the modified coefficients is computed by

$$d_{i}^{\prime }=\left[C_{\left(x,y\right)}-C_{i}^{\prime }\right]{|}_{\varphi =0,1,2}$$ (12)

where $C_{i}^{\prime }$ is the modified coefficient and (*i* = 1, 2) is related to the total number of modified coefficients. The obtained two difference values of $d_{i}^{\prime }$ are represent the decimal values of *ε*_(*D*)_ and *ε*_(*D*+1)_, $\left(E_{\left(D\right)}\;=\;d_{1}^{\prime },\;E_{\left(D+1\right)}\;=\;d_{2}^{\prime }\right)$. These values are then converted to binary code value and each represented by 1 bit in *ε*_*T*(*i*)_ block. Finally, after all the embedded bits blocks are retrieved, the bytes of the encrypted secret data are built back in *ε*_*T*_ and every byte is converted to represent a character to get the Ciphertext.

## Performance Evaluation

### Test Video Sequence

The system is simulated using MATLAB version 7.17.0.739. To facilitate our investigation, the reference implementation of MPEG-2 is used and applied with EBBD steganographic technique on 150 frames of each of the six widely known raw YUV video sequences in CIF format with 30fps and a resolution in pixels of 352 × 288 (width × height). All these video sequences are filmed using professional and high-end equipment to ensure that the reference sequences are alteration free. Furthermore, these sequences represent different combinations of motion and are classified into three classes based on their motion activities. The video sequences "Miss America" and "Suzie" contain still background with motion in the foreground and with limited motion in certain frames and such videos are classified as Slow-Motion Videos (SM-V). "Silent" and "Foreman" contain camera motion where the subject moves his/her entire upper body frequently twice toward the end of the video and are classified as Intermediate-Motion videos (IM-V).

The video sequences "Football" and "Stefan" contain camera panning and zooming along with considerable object motion and texture in the background. Therefore, a variation in motion of the background as well as the subject is observed, thereby, such videos are classified as Fast-Motion Videos (FM-V). Thus, the behaviour of the proposed system is analysed under various input characteristics. For the embedding payload, up to 4 KB of data is embedded and analysed on each of I-, P- and first B-frames of each of the 25 selected GOPs of the compressed video sequences.

### Performance Evaluation Parameters

The performance evaluation of the EBBD is done by examining the technique in terms of data hiding effects on the file size, invisibility and robustness. The performance of the proposed technique method is measured by comparing with existing steganographic techniques. Compression ratio (CR) has been identified as the number of bits of the original video divided by the number of bits used to represent the compressed video and is given in Eq (13). Peak signal to noise ratio (PSNR) metric is widely used to evaluate the quality of the video objectively and has been used to analyse the quality of the stego video is given in Eq (14).

$$CR=\frac{V_{s}}{V_{P}}$$ (13)

where *V*_*s*_ is the source video file size, *V*_*P*_ is the processed video file size

$$PSNR=10log_{10}\left(\frac{max(F^{2})}{MSE}\right)$$ (14)

Where MSE is determined through the following equation:

$$MSE=\frac{1}{MN}\sum_{m=1}^{M}\sum_{n=1}^{N}{\left(F_{m,n}-F_{m,n}^{\prime }\right)}^{2}$$ (15)

The mean square error (MSE) metric measures the difference between the reference and processed data signals. *F*_*m*,*n*_ is the original frame, and $F_{m,n}^{\prime }$ is the modified frame. *M* ×*N* is the size of the video frame. However, PSNR does not perfectly correlate with a perceived visual quality due to the non-linear behaviour of the human visual system (HVS).

Consequently, structural similarity index metric (SSIM) [24] is considered during the evaluation, given in Eq (16), because such metric has a better correlation to the subjective impression. The SSIM metric is based on structural content and estimates the similarity of two data signals by measuring the visible difference between distorted and reference data signals. The output of SSIM value is confined in the range between 0 and 1. If SSIM value is close to 1, then the distorted signal is of good quality.

$$SSIM(F,F^{\prime })=\frac{\left(2\mu_{F}\mu_{F^{\prime }}+C_{S1}\right)\left(2\sigma_{FF^{\prime }}+C_{S2}\right)}{\left(\mu_{F}^{2}+\mu_{F^{\prime }}^{2}+C_{S1}\right)\left(\sigma_{F}^{2}+\sigma_{F^{\prime }}^{2}+C_{S2}\right)}$$ (16)

where *μ* represents the mean intensity of the *F*_*m*,*n*_ and $F_{m,n}^{\prime }$, *C*_*S1*_ and *C*_*S2*_ are constants.

Robustness is measured by the bit error rate (BER) of the extracted embedded data as a function of the amount of distortion introduced by a given manipulation to determine the reliability of the extracted data. BER is defined as the ratio of incorrectly extracted data bits by the decoder side to the total number of embedded bits. BER is 0.00 for most extracted data, except for the exchange attack where it is larger. The robustness of the embedding methods is given in Eq (17)

Robustness=(1−BER)×100 (17)

## Results and Discussion

In order to evaluate the performance of the EBBD, data payload of 32768 bits has been tested on the selected video sequences. The original video streams are obtained by the MPEG-2 implemented software, and the embedding schemes are integrated with the MPEG-2 coder. All selected video sequences are encoded with MPEG-2, with their resolutions of 352×288 and 30fps. The evaluation was based on a sample of 25 GOP and picture ratio of I:P:B = 1:1:4 from each video (i.e. 6×25 = 150 frames).The following terminology is used in this section:. *CR*_*cover*_ and *CR*_*stego*_ are the compression ratios generated from the MPEG-2 cover video and stego MPEG-2 video, respectively. *VFS*_*cover*_ and *VFS*_*stego*_ are the video file sizes of the MPEG-2 cover video and stego MPEG-2 video, respectively.

### The Effect of Data Hiding on Video File Size

This section discusses the influence of embedding data on SM-V, IM-V, and FM-V MPEG-2 sequences when data payload of 32768 bits is embedded using EBBD. The recorded results are tabulated in Table 1 generated from MPEG-2 videos and stego videos utilized to summarize the efficiency of each selected technique versus video characteristics (video category) and frame type. On the sequence category basis, the summarized achievable relative *VFS*_*stego*_ increase and *CR*_*stego*_ reduction expressed in percentages is tabulated in Table 2 for each frame type.

**Table 1:** Data hiding results with the intra and the inter frames of the test video sequences for different steganographic techniques for the scenario that the data payload of 32768 bits are embedded in the MPEG-2 encoder.

Cat	Sequence	MPEG-2		Scheme	Stego MPEG-2					
		*VFS*_*cover*_ (MB)	*CR*_*cover*_		*VFS*_*stego*_ (MB)			*CR*_*stego*_		
					I	B	P	I	B	P
SM-V	Suzie	1.721	12.62	BPCS	1.83	1.82	1.82	11.86	11.92	11.92
				EBBD	1.88	1.88	1.88	11.54	11.54	11.54
				PVD	1.89	1.89	1.89	11.48	11.48	11.48
				EPVD	1.87	1.87	1.87	11.60	11.60	11.60
				TPVD	1.84	1.84	1.84	11.79	11.79	11.79
	Miss America	2.17	10	BPCS	2.28	2.27	2.27	9.52	9.56	9.56
				EBBD	2.31	2.30	2.30	9.39	9.43	9.43
				PVD	2.35	2.35	2.35	9.23	9.23	9.23
				EPVD	2.32	2.31	2.31	9.35	9.39	9.39
				TPVD	2.30	2.29	2.29	9.43	9.48	9.48
IM-V	Silent	2.96	7.33	BPCS	3	3.02	3.02	7.23	7.19	7.19
				EBBD	3.03	3.04	3.04	7.16	7.14	7.14
				PVD	3.07	3.12	3.11	7.07	6.96	6.98
				EPVD	2.99	3.04	3.04	7.26	7.14	7.14
				TPVD	3.06	3.08	3.07	7.09	7.05	7.07
	Foreman	3.45	6.29	BPCS	3.51	3.50	3.5	6.18	6.20	6.20
				EBBD	3.54	3.53	3.53	6.13	6.15	6.15
				PVD	3.56	3.58	3.57	6.10	6.06	6.08
				EPVD	3.51	3.54	3.54	6.18	6.13	6.13
				TPVD	3.53	3.54	3.54	6.15	6.13	6.13
FM-V	Football	4.55	4.77	BPCS	4.61	4.6	4.6	4.71	4.72	4.72
				EBBD	4.64	4.64	4.64	4.68	4.68	4.68
				PVD	4.68	4.68	4.67	4.64	4.64	4.65
				EPVD	4.63	4.61	4.6	4.69	4.71	4.72
				TPVD	4.65	4.65	4.64	4.67	4.67	4.68
	Stefan	5.69	3.81	BPCS	5.72	5.72	5.72	3.79	3.79	3.79
				EBBD	5.75	5.73	5.74	3.77	3.79	3.78
				PVD	5.74	5.75	5.75	3.78	3.77	3.77
				EPVD	5.71	5.71	5.71	3.80	3.80	3.80
				TPVD	5.73	5.74	5.73	3.79	3.78	3.79

*CR*_*cover*_, compression ratios generated fromMPEG-2 cover video; *CR*_*stego*_, compression ratios generated from stego MPEG-2 video; *VFS*_*cover*_, video file sizes of the MPEG-2 cover video; *VFS*_*stego*_, video file sizes of the stego MPEG-2 video.

**Table 2:** Percentage average alteration in video file size and compression ratio when I, B or P-frames are considered as a cover using techniques for the scenario that the data payload of 32768 bits are embedded in the MPEG-2 encoder.

Category	Scheme	Percentage increase (%) in *VFS*_*stego*_			Percentage decrease (%) in *CR*_*stego*_		
		I	B	P	I	B	P
SM-V sequences	BPCS	5.66	5.14	5.14	5.48	5.04	5.04
	EBBD	7.71	7.46	7.46	7.47	7.29	7.29
	PVD	9	9.00	9.00	8.44	8.44	8.44
	EPVD	7.71	7.46	7.46	7.38	7.21	7.21
	TPVD	6.43	6.17	6.17	6.19	5.97	5.97
IM-V sequences	BPCS	1.56	1.72	1.72	1.54	1.69	1.69
	EBBD	2.50	2.50	2.50	2.42	2.42	2.42
	PVD	3.43	4.52	4.21	3.30	4.41	4.11
	EPVD	1.40	2.65	2.65	1.32	2.57	2.57
	TPVD	2.81	3.28	3.12	2.79	3.23	3.08
FM-V sequences	BPCS	0.88	0.78	0.78	0.93	0.82	0.82
	EBBD	1.46	1.27	1.37	1.52	1.28	1.40
	PVD	1.76	1.86	1.76	1.86	1.98	1.86
	EPVD	0.98	0.78	0.68	1.05	0.82	0.70
	TPVD	1.37	1.46	1.27	1.40	1.52	1.28

As indicated in the Table 2, regardless of the embedding technique, the average alteration in the *VFS*_*stego*_ and *CR*_*stego*_ is less for FM-V sequences followed by IM-V and SM-V sequences in all embedding cases of the frame type because the magnitude of the AC-QTCs are not significantly altered by the embedded data that reduce the code length of the VLC. Hence, FM-V sequences are more applicable for data embedding because of the texture and high amount of motion within these sequences.

The Table 2 shows that among all the benchmark steganographic techniques, BPCS technique produces the lowest alteration (minimum alteration), whereas the PVD technique produces the highest. Although the PVD scheme utilized fewer QTC blocks compared with the BPCS technique, the alteration in the *VFS*_*stego*_ increased considerably. Such technique has a minimal modification in the AC-QTCs with magnitude zero because the data are mostly hidden in the bit-planes for the low and medium frequency components of the AC-QTCs.

Although the TPVD utilizes fewer QTCs, it has significantly altered these selected AC-QTCs, particularly for AC-QTCs with a magnitude of zero because a large number of data bits are embedded by changing the difference between the values of the selected coefficients. For the EBBD technique, regardless of the number of utilized blocks, the increase in the *VFS*_*stego*_ is mostly due to the use of the medium and high frequency components of the AC-QTCs. However, the EBBD scheme preserves the quality of the video sequence at the expense of the *VFS*_*stego*_ increment.

The EPVD performs better than its original version (PVD) because of the modification applied on the original PVD that always makes the absolute difference almost equal to the original two coefficient blocks, particularly for AC-QTCs with a magnitude of zero. However, EPVD utilizes more QTCs blocks compared with the PVD. Using the *VFS*_*stego*_ increased with the BPCS technique as the reference; the average increase of *VFS*_*stego*_ is 2.23%, 3.69%, 2.23%, and 0.95% for EBBD, PVD, EPVD, and TPVD, respectively, for the data embedded to the SM-V sequences case scenario. For the scenario of embedding data in IM-V sequences, the *VFS*_*stego*_ increase up to 0.83%, 2.38%, 0.56%, and 1.4% for EBBD, PVD, EPVD, and TPVD, respectively, and to 0.56%, 0.98%, 0.00%, and 0.56%, respectively, when the data are hidden in FM-V sequences. The level of alteration in the *VFS*_*stego*_ is basically based on the number of selected blocks, frequency component type (low, medium, and high frequency components), and the amount of data bits embedded in these blocks. According to the results, this effect can be almost ignored especially when the data are embedded into IM-V and FM-V sequences. The relationship between *CR*_*stego*_ and *VFS*_*stego*_ is non-linear and exponential. Thus, the *CR*_*stego*_ decreases as the *VFS*_*stego*_ increases, and vice versa.

### Imperceptibility Performance

**Fig 7:**
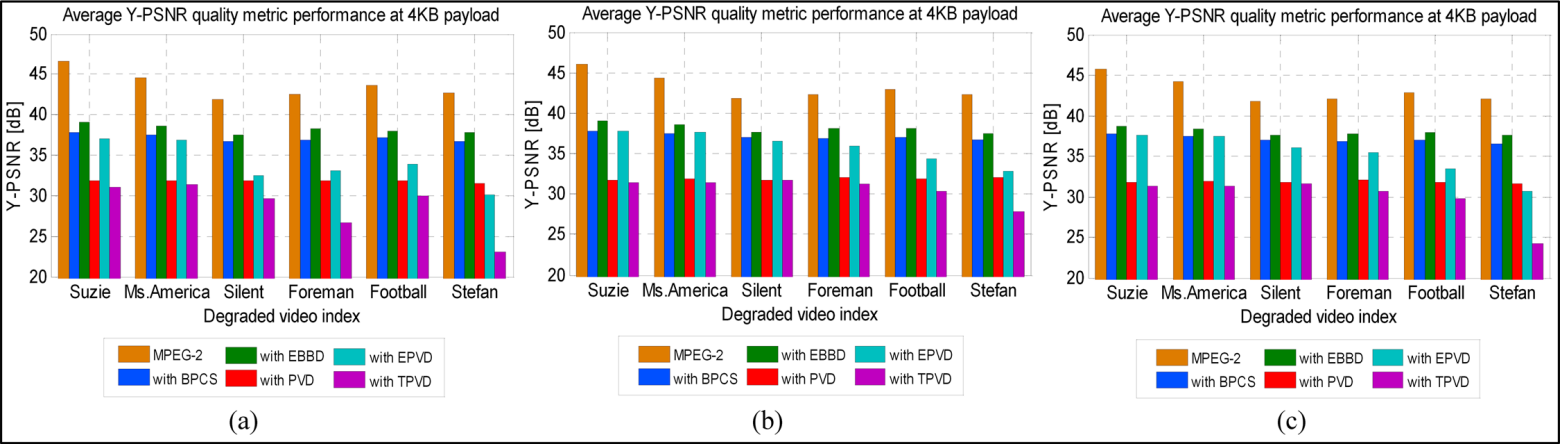
PSNR quality predicted values for Y-components of intra and inter frames for six stego videos sequences. (a) I-frame, (b) B-frame and (c) P-frame.

**Fig 8:**
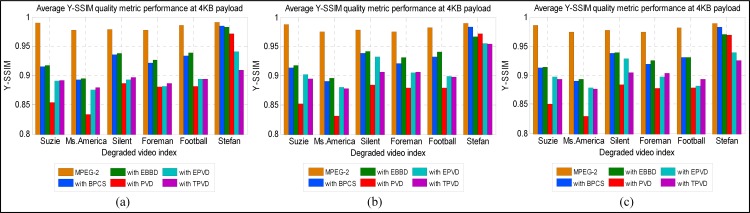
SSIM quality predicted values for Y-components of intra and inter frames for six stego videos sequences. (a) I-frame, (b) B-frame and (c) P-frame.

We evaluate the imperceptibility of the EBBD using PSNR and SSIM quality metrics. The secret data are hidden within the luminance component of the video sequence. Hence, the quality metrics are used to evaluate the quality of the stego video over the luminance channel only of the stego video (compressed + embedded) with respect to original video (compressed only). Figs 7 and 8 shows quality result values that are obtained through averaging the outcomes of the luminance component quality values for the I-, B-, and P-frames of six video sequences for the considered six methods. The average metrics values of the coded components with and without embedded data are given for the sake of comparison.

Fig 7(A)–7(C) shows that among the embedding schemes, EBBD performs best, using the Y-PSNR of the unmodified video (compressed without embedded data) as reference, on a frame basis. The average decrease in Y-PSNR for EBBD in the overall video sequences is around 5.45, 5.17, and 5.10 dB when secret data are embedded in I-, B-, and-P frames. Each selected QTC will either be the same or modified by ±1 depending on whether the bit to be embedded is “0” or “1”. Therefore, less modification to QTCs results in less degradation in the quality. A higher decrease in Y-PSNR is observed for the TPVD scheme. The average decrease in Y-PSNR is around 15, 12, and 13.28 dB for the same frame types. The TPVD allows a larger data payload to be embedded, which causes significant changes in the magnitudes of the QTCs, especially for those with a zero magnitude coefficient, which will eventually affect the overall stego sequence quality seriously. Similar observations are reported for the EPVD and PVD schemes. An average decrease in the Y-PSNR for EPVD is approximately 9.74, 7.45, and 8.02 dB for I-, B-, and P-frames, while it is approximately 11.9, 11.41, and 11.32 dB for the same frame types when PVD is utilized.

EPVD outperforms the PVD scheme. This finding implies that modifications applied on the original PVD, which makes the absolute difference as close to the original two-coefficient block as possible, especially for AC-QTCs with a zero magnitude, thereby successfully reducing the degradation in the video quality caused by the PVD scheme. However, these modifications preserve the quality of the video at the expense of embedding payload. To evaluate stego video quality, SSIM metric, which is believed to be more consistent with HVS perception than previous metrics, is employed to evaluate the perceptual quality of each test video sequence. According to its definition, a larger SSIM value corresponds to higher visual quality. To demonstrate the quality performance for video sequences individually, Fig 8(A)–8(F) depicts Y-SSIM comparisons as functions of video sequence indices for secret data embedding in I-, B-, and P-frames. Steganographic scheme performances vary based on the type of video and utilized frame overall video sequences.

On the frame basis, among the steganographic schemes, EBBD generates better-quality stego videos with higher Y-SSIM values compared with other schemes (less reduction in Y-SSIM), while PVD generates the worst quality stego videos with lower Y-SSIM values. EBBD demonstrates better performance. An average decrease in Y-SSIM is 0.050, 0.049, and 0.051 dB for I-, B-, and P-frames, respectively, is observed for EBBD, while a similar average reduction of 0.099 dB for I-, B-, and P-frames is observed for PVD. BPCS performs worse than EBBD, followed by EPVD, and TPVD. For I-, B-, and P-frames, the average decrease is 0.053, 0.051, and 0.052 dB, respectively, for BPCS, 0.058, 0.056, and 0.057 dB, respectively, for EPVD, and 0.091, 0.075, and 0.081 dB, respectively, for TPVD.

**Fig 9:**
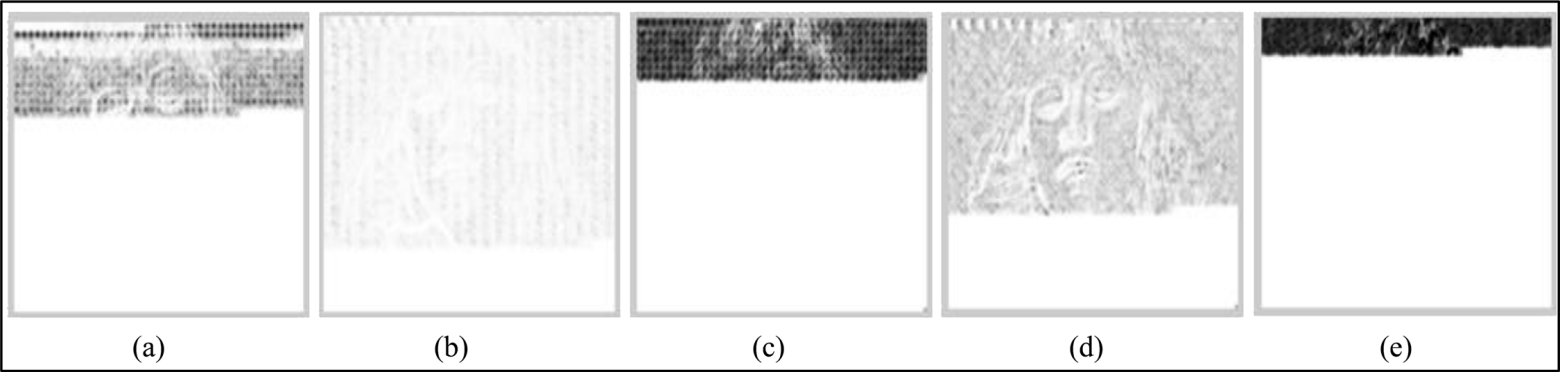
Samples of SSIM quality map for Y-components of the I-frame within the 3^rd^ GOP of the stego MPEG-2 "Suzie" using different steganographic methods. (a) BPCS, (b) EBBD, (c) PVD, (d) EPVD and (e) TPVD.

**Fig 10:**
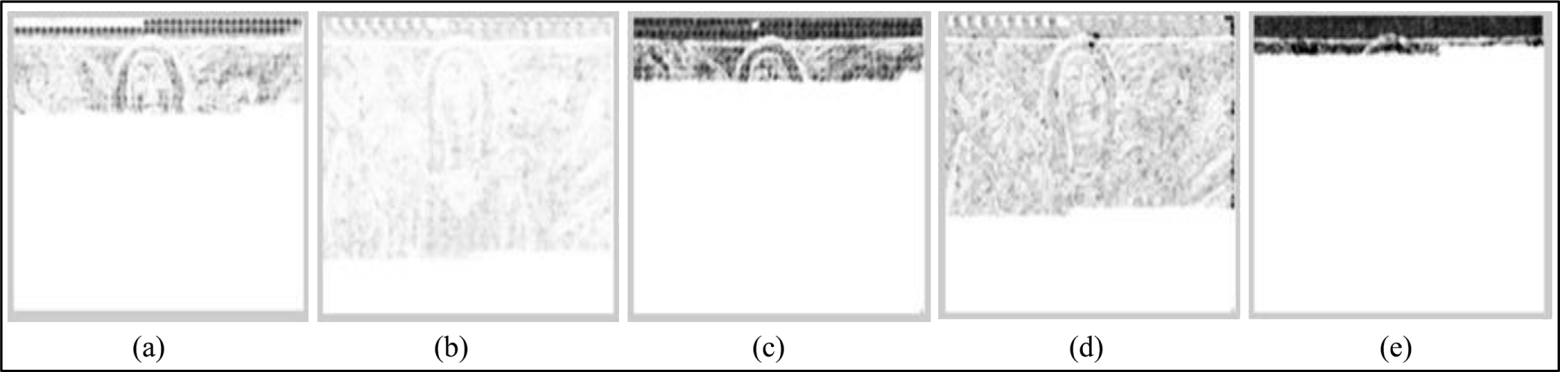
Samples of SSIM quality map for Y-components of the I-frame within the 3^rd^ GOP of the stego MPEG-2 "Silent" using different steganographic methods. (a) BPCS, (b) EBBD, (c) PVD, (d) EPVD and (e) TPVD.

**Fig 11:**
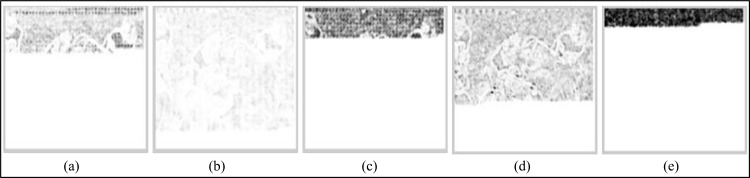
Samples of SSIM quality map for Y-components of the I-frame within the 3^rd^ GOP of the stego MPEG-2 "Football" using different steganographic methods. (a) BPCS, (b) EBBD, (c) PVD, (d) EPVD and (e) TPVD.

On a video category basis, among the different data embedding schemes, EBBD performs best, having a minimum average decrease in video quality for all types of video categories (i.e. SM-V, IM-V and FM-V). Samples of the SSIM error maps of the Y-components of the intra stego frame within the third GOP for the “Suzie,” “Silent,” and “Football” are given in Figs 9–11. The SSIM map is more consistent with observations related to the imperceptibility performances of stego video sequences for all embedding schemes. Generally, brighter blocks (SSIM values are closer to 1) correspond to a higher similarity between the stego coded frame and its corresponding coded frames without data embedding. From these figures, an evident observation based on the maps is that EBBD performs best among all embedding schemes (Y-SSIM is 0.92018, 0.93714 and 0.95965 for "Suzie," "Silent," and "Football," respectively).

Maps are significant for the BPCS scheme especially for the top left side of the frames, as shown in Figs 9(A) and 10(A) for the "Suzie," and "Silent" video sequences where their corresponding Y-SSIM values are 0.89508, and 0.91581, respectively while the map is less significant for "Football" sequence, as shown in Fig 11(A), (Y-SSIM = 0.93410). Intra frames generated by PVD and TPVD are heavily distorted compared to other modified version of PVD (i.e. EPVD), and their SSIM values are 0.88276 and 0.89541 on average for the three test sequences.

### Visual Quality Performance

The subjective evaluation of the stego videos is based on determining the difference in quality between the coded versions with MPEG-2 codec without data embedding, and stego MPEG-2 coded versions generated using different embedding techniques. Similar observations are provided to the same class videos for SM-V, IM-V and FM-V, respectively. To evaluate the visual quality performances of stego video sequences generated from different embedding techniques, instead of being concentrated in a relatively small area of the frame, for a given scene, backgrounds of video frames can be considered.

Among the embedding schemes, EBBD clearly performs better. No perceivable degradation in the visual quality in the case of intra and inter frames exist compared with corresponding MPEG-2 coded frames without data embedding for all of the SM-V, IM-V and FM-V video sequences. This finding implies that increasing the embedded payload may be possible using the proposed EBBD technique before any perceivable effect occurs on the characteristics of the encoded video.

Using BPCS, small encircled white artifacts are found around the left side of the person and the person’s facial hair, and more artifacts are visible in white and black spots on the left for the SM-V video sequences. However, such artifacts slightly appeared in the IM-V video sequences, except those artifacts around the person’s face, which is considerably visible because of the background color. Blotch artifacts are more apparent around the players for the "Football" and even in "Stefan" sequences.

The strongly amplified difference between the stego frames generated using PVD and the corresponding coded frames is observed for each video within each video category sequences. Artifacts can be observed in the stego frames because of data embedding especially for the backgrounds of video frames. Artifacts are visible as blurred edges in some blocks and blotches. Blotches appear because of the significant change in luminance and drift effect. However, these artifacts are hardly found in the same stego frames generated using the proposed EPVD for all of the SM-V, IM-V and FM-V video sequences. This finding implies that modifications applied to the original PVD, which makes the absolute difference as close as possible to the original two coefficient block, especially for AC-QTCs with magnitude zero, successfully reduces the artifacts caused by PVD in the video. The visual quality of intra frames of the same class videos for SM-V, IM-V and FM-V are severely degraded. In the stego frame, fewer AC-QTCs were employed compared with that in the PVD scheme, but it was heavily modified. Therefore, the noise is due to the introduction of new QTCs because of serious conversion especially for zero AC-QTCs.

### Robustness Performance

Table 3 presents a summary of BER values of different steganographic techniques for the conducted compression attack. BERs are measured in case data are embedded into intra frames of the tested video sequences. The results show that EBBD is always better than others with BERs of 0.00. Using the Eq (17) below to determine the robustness of the embedding schemes, the EBBD is most robust against compression. On average, achievable robustness of 100%, 100%, and 99.998% are observed for SM-V, IM-V, and FM-V video sequences, respectively.

**Table 3:** Summary of BER values of different steganographic techniques for compression attack.

Sequence	BPCS	EBBD	PVD	EPVD	TPVD
Suzie	0	0	0	0	0.082332026
Miss America	0.000183914	0	0.014222658	9.20E-05	0.094868808
Silent	3.07E-05	0	0.072860471	0.000490436	0.360194949
Foreman	5.52E-03	0	0.036261648	0.00309588	0.094868808
Football	3.07E-05	0	0.47768514	0.137659392	0.087818784
Stefan	0.008368073	3.07E-05	0.380057626	0.295917116	0.383521334

## Conclusion

A new secured video steganography technique namely EBBD has been proposed in the compressed domain that has the capability to preserve the video quality. The EBBD technique can embed certain of data and compress video to MPEG-2 at the same time. In addition, the EBBD technique has been introduced to improve the embedding efficiency by trading off with payload. The technique embeds the encrypted data bits in the AC-QTCs for different types of frames. Selection of appropriate QTCs blocks, the embedding algorithm, and use of a pseudo random key enhanced security of the proposed technique without adversely affecting the overall file size, SSIM and the PSNR of the video bitstream. The validation of the technique was performed by a series of experiments that proved the ability of the model to embed encrypted hidden data into luminance components for both intra and inter-frames for a variety MPEG-2 compressed video sequences and was compared with five recently reported data hiding schemes [16, 20, 21, 22] under the same conditions. Results of the experiments are positive and provide a base for further research. The performance of the proposed EBBD technique is more efficient in practice, demonstrating that such technique provides optimal balance between invisibility, embedding capability and security. The obtained PSNR values are close to the reference compressed video (compressed video without data hiding) and the SSIM is close to 1, taking into account that the SSIM index is a good indicator of the perceptual quality.

The success of the technique also highlights the usefulness of experimental approach in solving the problem of secure communication. Although data to be embedded has been encrypted using S-DES algorithm, for additional layer of security, an encryption key based shuffling is used to pseudo-randomly shuffle the host coefficients in each candidate block within a selected frame respectively. As result, it is virtually impossible for unauthorized people who know the algorithm to pirate the hidden information, without knowledge of the encryption key. In addition, the robustness of the EBBD has been evaluated against compression attack and it is found that it can resist such manipulation with robustness percentage close to100% on average.
